# Ultra-Low Dose of Superparamagnetic Iron Oxide Nanoparticles for Sentinel Lymph Node Detection in Patients with Breast Cancer

**DOI:** 10.1245/s10434-023-13722-x

**Published:** 2023-06-14

**Authors:** Nushin Mirzaei, Fredrik Wärnberg, Pontus Zaar, Henrik Leonhardt, Roger Olofsson Bagge

**Affiliations:** 1 Department of Surgery, Sahlgrenska Centre for Cancer Research, Institute of Clinical Sciences, Sahlgrenska Academy, University of Gothenburg, Gothenburg, Sweden; 2 Department of Surgery, Sahlgrenska University Hospital, Gothenburg, Sweden; 3 Wallenberg Centre for Molecular and Translational Medicine, University of Gothenburg, Gothenburg, Sweden; 4 Department of Radiology, Sahlgrenska University Hospital, Institute of Clinical Sciences, Sahlgrenska Academy at University of Gothenburg, Gothenburg, Sweden

**Keywords:** Breast cancer, Sentinel lymph node biopsy, Superparamagnetic iron oxide nanoparticles

## Abstract

**Background:**

Sentinel lymph node (SLN) status is pivotal for treatment decision-making in patients with breast cancer. Superparamagnetic iron oxide nanoparticles (SPIO) have been shown to be equivalent to the dual technique with technetium^99m^ (Tc^99^) and blue dye (BD) for SLN detection. The aim of this study was to determine the feasibility of detecting SLNs using an ultra-low dose of SPIO.

**Method:**

Patients planned for breast conserving surgery and SLN biopsy were included. An intradermal injection of 0.1 mL SPIO was administered at the areolar border up to 7 days before surgery. Tc^99^/BD was administered according to clinical routine. SLNs were detected during surgery using a handheld magnetometer. All nodes with a magnetic and/or radioactive signal, as well as blue or clinically suspicious nodes, were harvested and analyzed.

**Results:**

In 50 patients, SPIO was injected a median of 4 days before surgery. At least one SLN was found in all patients with both methods. A total of 98 SLNs were removed; 90 were detected using SPIO and 88 using Tc^99^/BD. Of the 90 SLNs detected by SPIO, 80 were Tc^99^/BD positive (concordance 89%). Histopathological analysis classified 16 patients with tumor cells deposit and 9 with macro-metastasis > 2mm, where one SLN was identified only by the radioactive technique and one only by the magnetic technique.

**Discussion:**

SLN detection using 0.1 mL ultra-low dose SPIO injected intradermally was successful in all patients. A future analysis will determine whether the approach using an ultra-low dose of SPIO injected intradermally will minimize skin staining and MRI artefacts.

Breast cancer is the fifth leading cause of cancer deaths worldwide, with 2.26 million new patients in 2020.^1^ The sentinel lymph node (SLN) is the first lymph node that receives lymphatic drainage from a primary breast tumor site. SLN biopsy (SLNB) is an established technique for tumor staging in patients with breast cancer.^2^ The SLN is often identified using a radioactive isotope tracer (technetium^99m^, Tc^99^) and a blue dye (BD), patent blue V^®^. The SLN is then detected with a handheld gamma probe, and the BD helps to visualize the SLN during surgery.

There has been increasing interest in using superparamagnetic iron oxide nanoparticles (SPIO) for SLN identification, with several studies showing similar detection rates with SPIO to Tc^99^/BD.^3–9^ The current recommendation is to inject 1–2 mL of SPIO interstitially intra/peritumorally, or behind the areola, and the SLNs are then detected during surgery with a handheld probe (Sentimag^®^) that measures the strength of the magnetic field created by the SPIO.^4–10^ The iron deposited in the lymph node also stains the SLN brown, facilitating visualization during surgery.

While SPIO have been shown to be non-inferior to Tc^99^/BD for detecting SLNs, skin staining at the injection site^5–9^ and MRI artefacts in the breast^11–14^ have been raised as concerns. The extent of skin staining and MRI artefacts is correlated with SPIO dose and also depends on the injection site.^15–18^ In a randomized study, Rubio et al. reported significantly less skin staining with 1 mL SPIO compared with doses of 1.5 mL and 2.0 mL.^8^ An even lower dose of SPIO could potentially minimize skin staining and MRI artefacts. In a study including patients with cutaneous melanoma, an ultra-low dose of 0.1 mL SPIO injected intradermally was feasible for detecting SLNs.^6^ The aim of this study was to evaluate the feasibility of using ultra-low dose SPIO injected intradermally for SLN detection in patients with breast cancer.

## Patients and Methods

### Study Population and Design

In this phase II, dose-escalation, single-arm study, women with histologically confirmed, clinically node-negative breast cancer planned for breast-conserving surgery and SLN biopsy were included. Exclusion criteria were age < 18 years; pregnancy; breastfeeding; hypersensitivity to iron, dextran compounds, or BD; and iron overload disease. A dose escalation schedule of SPIO (Magtrace^®^, Endomagnetics Ltd, Cambridge, UK) was planned: 0.1 mL, 0.25 mL, and 0.5 mL, with a minimum of five patients per step. If four or more SLNB procedures were successful, no further dose escalation was planned. The Swedish Ethical Review Authority (dnr: 2021-02726) and the Swedish Medical Product Agency (dnr: 5.1-2021-41266) approved the study, and the study was registered at clinicaltrials.gov (NCT05359783). All patients provided signed informed consent.

### SPIO, Tc^99^, and BD Tracer Injections

**Fig. 1:**
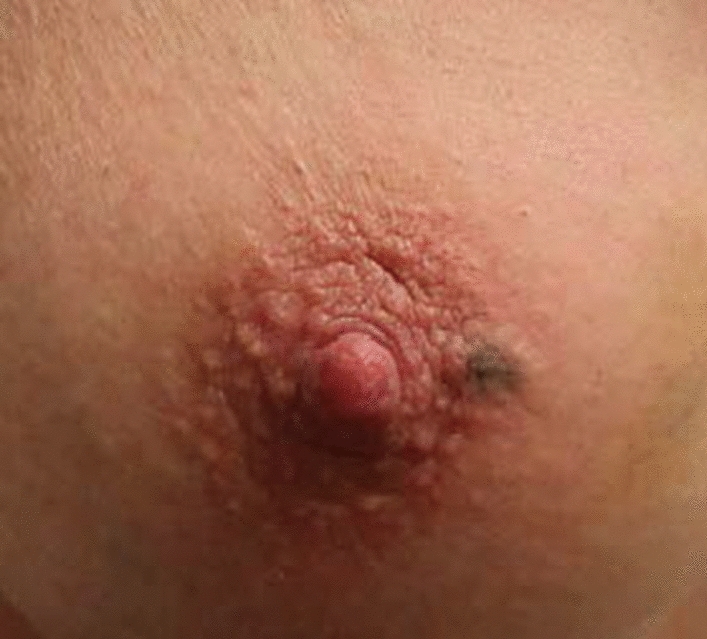
Preoperative skin staining after 0.1 mL intradermal SPIO injection at the outpatient clinic

Intradermal injections of SPIO were administered at the place of incision, or at the areolar border, 1–7 days prior to surgery to allow for easy removal of the small injection (Fig. 1). Tc^99^ and BD were injected according to routine clinical practice: Tc^99^ either on the day of or the day before surgery and BD after the onset of anesthesia.

### Surgery

The primary approach to identify SLNs at surgery was with the magnetic technique, with Tc^99^/BD used as a backup. Before the skin incision, magnetic and radioactive counts were registered transcutaneously over the injection site in the breast and in the axilla. Magnetic and radioactive counts were registered in vivo and ex vivo for every SLN removed. Lymph nodes containing more than 10% activity compared with the maximum activity of the highest scoring lymph node, for both the SPIO and Tc^99^, were considered SLNs. Each SLN was sent separately for routine histopathological assessment.

### Statistical Analysis

The main aim of this study was to evaluate the feasibility of identifying the SLN using ultra-low dose SPIO, and the results are reported descriptively with means and standard deviations (SD) and medians and interquartile ranges (IQR). Concordance was determined as the number of SLNs positive for both SPIO and Tc^99^/BD divided by the number of SPIO-positive SLNs. Reverse concordance was defined as the number of SLNs positive for both Tc^99^/BD and SPIO divided by the number of Tc^99^/BD-positive SLNs.

## Results

A total of 58 patients were screened for inclusion. One patient did not meet the inclusion criteria and seven patients declined to participate. In total, 50 women were included between November 2021 and January 2023. Their median age was 63 years (range 41–82 years), and the median tumor size was 14 mm (IQR 10–19 mm) (Table 1). SPIO was injected a median of 4 days before surgery (range 1–9 days). Due to the COVID-19 pandemic, one patient was rescheduled for surgery 2 days later than the 7-day upper limit specified in the protocol. As the first four patients had a successful SLNB procedure with 0.1 mL SPIO, no dose escalation was performed, and all patients in the study received 0.1 mL SPIO. All patients received breast-conserving surgery.

**Table 1:** Patient and tumor characteristics

Age, median (range)	63 (41–82) years
BMI*, median (IQR)	25 (24–29) kg/m^2^
Sex	
Female	50 (100%)
Tumor size, median (IQR)	14 (10–19) mm
Days between injection and surgery, median (range)	4 (1–9)
Tumor location	
Upper outer quadrant	19 (38%)
Upper inner quadrant	20 (40%)
Lower outer quadrant	7 (14%)
Lower inner quadrant	4 (8%)
Histological type	
Invasive ductal cancer	35 (70%)
Invasive lobular cancer	10 (20%)
Invasive tubular cancer	5 (10%)
Histopathological grade**	
1	11 (22%)
2	36 (72%)
3	3 (6%)
Receptor status	
ER^+^ HER2^−^	49 (98%)
ER^−^ HER^−^	1 (2%)

*IQR* interquartile range, *BMI* body mass index, *ER* estrogen receptor,

*HER2* human epidermal growth factor receptor 2

*Data are missing for one patient

**Grade according to Elston–Ellis

The SLNB procedure using SPIO and Sentimag^®^ was successful in all 50 patients (100%), and no adverse effects of SPIO were reported. Due to technical malfunction, the gamma probe was not available during one of the procedures, but in all 49 patients where the gamma probe was used, at least one SLN was detected with Tc^99^/BD and gamma probe (100%).

A total of 98 SLNs were removed, of which 90 were detected by SPIO, with a mean of 1.8 ± 1.1 [median 1 (IQR 1–2)] SLNs per patient. Of the 90 SLNs detected by SPIO, 80 were also Tc^99^/BD positive, giving a concordance of 89%. Of the total 98 SLNs removed, 88 were positive for Tc^99^/BD, with a mean of 1.7 ± 1.0 [median 1 (IQR 1–2)] SLNs per patient: 83 were only radioactive, 4 were radioactive and stained blue, and 1 node was stained blue without any radioactive (or magnetic) signal. Of the 88 SLNs detected using Tc^99^/BD, 80 were SPIO positive, giving a reverse concordance of 91%. The mean number of SLNs retrieved per patient were 2.0 ± 1 [median 1 (IQR 1–3)+ for SPIO combined with Tc^99^/BD. After histopathological analysis, 26 additional non-SLNs were identified, giving a total of 124 (98 + 26) lymph nodes analyzed.

In 13 patients (26%), no axillary transcutaneous magnetic signal was detectable before skin incision, but in all patients, including these 13 patients, a clear magnetic signal was detected after skin incision. The median ex vivo magnetic counts for the highest scoring SLN per patient using Sentimag^®^ was 1255 (IQR 242–3219), and the median ex vivo radioactivity counts using the gamma probe was 279 (IQR 27–973).

The histopathological analysis showed 16 patients having lymph node with tumor cells deposits: 9 patients with at least one macro-metastasis (≥ 2 mm), 5 patients with at least one micro-metastasis (< 2 mm), and 2 patients with isolated tumor cells (ITCs). Of the 98 removed SLNs, 18 contained tumor cells: ten macro-metastases, five micro-metastases, and three ITCs (Table 2). In one patient the macro-metastatic SLN was only identified by the radioactive technique, and in one patient the macro-metastatic SLN was only identified by the magnetic technique. Of the 26 non-SLNs, three nodes contained macro-metastasis, all in the same patient where two other SLNs were both SPIO and Tc^99^/BD positive. In summary, one patient would be falsely classified as node negative if only using SPIO and another if only using Tc^99^/BD. Furthermore, another patient with metastatic SLN would potentially have been falsely classified, as the node was only stained blue without a radioactive signal and had a positive magnetic signal.

**Table 2:** Summary of removed sentinel lymph nodes

Patients *n* = 50	Total	SPIO-positive	Tc^99^/BD-positive
Successful SLNB, *n*		50/50 (100%)	49/49 (100%)*
SLNs retrieved, *n*	98	90/98 (92%)	88/97 (91%)*
SLNs with tumor cells, *n*	18	17/18 (94%)	16/17 (94%)*
Macro-metastatic SLNs, *n*	10	9/10 (90%)	9/10 (90%)
Micro-metastatic SLNs, *n*	5	5/5 (100%)	5/5 (100%)
ITC SLNs, *n*	3	3/3 (100%)	2/2 (100%)*

*The gamma probe was not available for one patient, and therefore Tc99 data are missing for one patient

## Discussion

In this prospective single-arm feasibility study, it was shown that SLN detection is feasible with an ultra-low dose of 0.1 mL SPIO injected intradermally in patients with breast cancer. This approach achieved a per patient SLN detection rate of 100%. The mean number of SLNs detected with 0.1 mL SPIO injected 1–9 days before surgery, compared with Tc^99^/BD injected according to routine practice, were similar. Furthermore, one patient per each method would have been falsely classified as node negative, indicating that the magnetic technique is as safe as the radioactive technique for identifying metastatic SLNs, even at low doses.

Using SPIO for SLNB identification in breast cancer has attracted interest since Thill et al. first reported the approach in 2014, and is now becoming a viable alternative to Tc^99^/BD.^4–7,10,19^ SPIO has several logistical advantages: the nanoparticles are detectable several days and weeks after administration and the approach does not require the same advanced facilities as for Tc^99^ detection. Furthermore, while BD can cause allergic reactions, no such reactions have been reported with SPIO.^19,20^

Nevertheless, there are some concerns with SPIO, such as potential skin staining and MRI artefacts within the breast that may cause diagnostic difficulties.^4,15–18^ Using a lower SPIO dose could potentially overcome these problems, especially if the injection is made at the place of incision where it can easily be removed.^6–8,10^ Rubio et al. investigated three different SPIO (Sienna XP) doses (2.0, 1.5, and 1.0 mL) in the SUNRISE trial and found that a low dose of 1.0 mL was not inferior to a higher dose of SPIO, nor to the conventional technique (Tc^99^/BD), for SLN detection in patients with breast cancer. There was also significantly less skin staining at 1- and 6-month follow-ups in patients who received 1.0 mL SPIO.^8^

Hersi et al. compared three different doses injected during different timeframes. The first cohort consisted of patients receiving 1.0 mL SPIO injected in subareolar or peritumoral areas 1–7 days before surgery; patients in the second cohort received 1.5 mL SPIO injected on the day of surgery; and the third cohort were patients from the Nordic SentiMag trial receiving 2.0 mL diluted with 3.0 mL of saline injected in the subareolar area, either shortly before or after onset of anesthesia.^5,10^ The results showed that the lower dose of 1.0 mL injected 1–7 days before surgery had a non-significantly higher detection rate (100%) than the 1.5 mL (97.5%) and 2.0 mL (97.6%) doses, indicating that the timing of injection could be important.

In a systematic review, Zada et al. analyzed seven studies comparing 2.0 mL SPIO with Tc^99^/BD administrated as a subareolar or periareolar subcutaneous injection. They reported a mean LN retrieval rate per patient of 2.1 for SPIO combined with Tc^99^/BD, 1.9 for SPIO alone, and 1.8 LNs for Tc^99^/BD alone.^21^ These findings are similar to our results, as the mean number of SLNs retrieved per patient was 2.0 for SPIO combined with Tc^99^/BD, 1.8 nodes for SPIO alone, and 1.7 nodes for Tc^99^/BD alone, despite the significantly lower dose of SPIO injected.

This study has some limitations. It is a single-center trial, potentially affecting the external validity and generalizability. Furthermore, there was no randomization, and the sample size was relatively small. However, our aim was to demonstrate the feasibility for SLN detection with an ultra-low dose of SPIO in patients with breast cancer, the results are convincing, and no dose escalation beyond 0.1 mL SPIO was necessary. In a preplanned analysis of this cohort after 12 months of follow-up, skin staining and postoperative MRI artefacts will be analyzed and reported separately.

In summary, SLN detection is feasible with an ultra-low dose of 0.1 mL SPIO injected intradermally in patients with breast cancer. However, further studies are needed to fully establish the clinical validity of this dose, and to verify that the ultra-low dose reduces skin staining and MRI artefacts.
